# Acute stress alters individual risk taking in a time‐dependent manner and leads to anti‐social risk

**DOI:** 10.1111/ejn.13395

**Published:** 2016-09-23

**Authors:** S. Bendahan, L. Goette, J. Thoresen, L. Loued‐Khenissi, F. Hollis, C. Sandi

**Affiliations:** ^1^ Laboratory of Behavioral GeneticsBrain Mind InstituteEcole Polytechnique Fédérale de Lausanne (EPFL)CH‐1015LausanneSwitzerland; ^2^ Department of EconomicsFaculty of Business and EconomicsUniversity of Lausanne (UNIL)CH‐1015LausanneSwitzerland; ^3^ Present address: Department of EconomicsInstitute for Applied MicroeconomicsUniversity of BonnAdenauerallee 24–42D‐53012BonnGermany

**Keywords:** behavioural economics, behavioural neuroscience, decision‐making, resilience, stress

## Abstract

Decision‐making processes can be modulated by stress, and the time elapsed from stress induction seems to be a crucial factor in determining the direction of the effects. Although current approaches consider the first post‐stress hour a uniform period, the dynamic pattern of activation of the physiological stress systems (i.e., the sympathetic nervous system and hypothalamic‐pituitary‐adrenal axis) suggests that its neurobehavioural impact might be heterogeneous. Here, we evaluate economic risk preferences on the gain domain (i.e., risk aversion) at three time points following exposure to psychosocial stress (immediately after, and 20 and 45 min from onset). Using lottery games, we examine decisions at both the individual and social levels. We find that risk aversion shows a time‐dependent change across the first post‐stress hour, evolving from less risk aversion shortly after stress to more risk averse behaviour at the last testing time. When risk implied an antisocial outcome to a third party, stressed individuals showed less regard for this person in their decisions. Participants’ cortisol levels explained their behaviour in the risk, but not the antisocial, game. Our findings reveal differential stress effects in self‐ and other‐regarding decision‐making and highlight the multidimensional nature of the immediate aftermath of stress for cognition.

## Introduction

Exposure to stressful situations triggers the activation of physiological and neuropsychological responses – particularly “fight‐or‐flight” responses – that have been selected throughout evolution for their ability to facilitate coping with life threats (McEwen, 2007). The physiological stress responses comprise the rapid (and generally transient) activation of the sympathetic nervous system (SNS), closely followed by activation of the hypothalamic‐pituitary‐adrenal (HPA) axis (Herman *et al*., 2012), whose actions can target the brain and affect ongoing and subsequent behavioural and cognitive functions (Roozendaal & McGaugh, 2011; Hermans *et al*., 2014), including social behaviours (Sandi & Haller, 2015).

It is therefore not surprising that decision‐making processes are susceptible to modulation by stress [for reviews, see (Starcke & Brand, 2012; Morgado *et al*., 2015)]. In our society, stress is rather ubiquitous in contexts where people are required to make important economic, social or political decisions. Decisions can vary in their targets; e.g., they can primarily affect the decision‐making agent, other individuals, or both. Acute stress appears to modulate decisions related to these different targets. For self‐related decisions, the emerging picture outlines risky decisions for gains – not losses (but see Pabst *et al*., 2013a, b; Robinson *et al.,* 2015) – as being particularly affected by stress (Lighthall *et al*., 2009; Porcelli & Delgado, 2009; Buckert *et al*., 2014); however, there is no consensus as to whether stress has any effect at all (Lempert *et al*., 2012; Gathmann *et al*., 2014); or turns individuals less (Lighthall *et al*., 2009; Buckert *et al*., 2014) or more (Porcelli & Delgado, 2009) risk averse. Similarly, evidence is mixed regarding the effects of acute stress in other‐regarding decisions. While some reports underscore prosocial effects of stress (von Dawans *et al*., 2012; Margittai *et al*., 2015), others describe antisocial effects (Vinkers *et al*., 2013; FeldmanHall *et al*., 2015; Margittai *et al*., 2015; Steinbeis *et al*., 2015). These discrepancies may be accounted for by different factors, such as gender effects (Preston *et al*., 2007; van den Bos *et al*., 2009; Lighthall *et al*., 2009), individual differences in hormonal stress responses (Coates & Herbert, 2008; Starcke *et al*., 2011; van den Bos *et al*., 2013b; Buckert *et al*., 2014; Kandasamy *et al*., 2014; Cueva *et al*., 2015), or the nature of stressors (Steinbeis *et al*., 2015).

Importantly, recent evidence suggests that the time elapsed from stress induction to behavioural testing is a crucial factor in capturing the effects of stress on decision‐making (Pabst *et al*., 2013a; Vinkers *et al*., 2013; Margittai *et al*., 2015). Several studies have focused on the distinction between two temporal domains (Joels & Baram, 2009): the first one taking place during the first hour after stress and the second lasting for several hours afterwards (Vinkers *et al*., 2013; Margittai *et al*., 2015). These phases are based on different mechanisms elicited by glucocorticoids (primarily cortisol in humans), the final products of the HPA axis that involve non‐genomic, rapid actions in the first phase and slower, genomic actions in the second with each of them engaging divergent activation of large‐scale brain networks (Joels *et al*., 2011; Hermans *et al*., 2014). So far, changes in decision‐making were found during the first but not the second phase (Vinkers *et al*., 2013; Margittai *et al*., 2015), highlighting the first post‐stress hour as the critical period for immediate stress effects on risk taking.

From an evolutionary point of view, the sensitivity of the first hour in the aftermath of stress to changes in risk aversion makes sense as it arguably corresponds with the period when responses to encountered threats are most needed. However, this first post‐stress hour may not be a uniform period with regards to decision‐making, but one that varies with time, as suggested by a recent study in which decisions changed at different time points throughout the hour (Pabst *et al*., 2013a). This view aligns well with the different physiological states experienced during this period, as exemplified by the dynamic pattern of activations elicited by acute stress on the SNS (very rapid and transient) and the HPA axis (typically, glucocorticoid levels arise slowly and peak at around 15–30 min post‐stress onset followed by a slow decline over the subsequent 30 min period). Therefore, we hypothesized that exposure to psychosocial stress would decrease risk aversion and lead to anti‐social decision‐making, with effects varying at discrete time points throughout the first post‐stress hour.

We set this study to investigate these hypotheses regarding risky economic decision‐making in the gain domain for self‐ and other‐regarding decisions, and explicitly asked whether stress effects would vary across different time points within the first hour following stress exposure. Given the lack of information, we did not make specific predictions for each time point. We used two economic games given to different cohorts of control and stressed participants at different time points within the first hour after stress induction. Although a marked bias towards risk aversion has been observed in several species, including humans, both individual differences and intra‐individual changes in risk taking have been documented (Markowitz, 1952; Kahneman & Tversky, 1979). We included males and females to test for gender effects, and measured heart rate and saliva cortisol levels to, respectively, assess SNS and HPA axis responses.

## Materials and methods

### Participants

Healthy male and female participants were recruited at the University of Lausanne and Ecole Polytechnique Fédérale de Lausanne (EPFL). Exclusion criteria included current medication usage; pregnancy, or breastfeeding; experiencing a major life change or an unusual amount of stress; smoking more than five cigarettes per day; having a history of medical or psychiatric illness, insomnia, night shift work or a history of drug or alcohol abuse. Three separate experimental blocks were conducted. Participants completed sessions in groups of five or six. The final sample size was 352 participants, randomly assigned to either stress (*n* = 173: 67 females and 106 males) or control (*n *=* *179: 75 females, 104 males) conditions. Sessions took place daily either between 14:00 and 16:00 or between 16:00 and 18:00. We conducted one stress and one control session on each day, with session order counterbalanced across experiment days.

Participant demographics are listed in Table 1. An additional group of 55 participants was recruited separately to play the role of second movers. These volunteers did not make any decisions, but received a cash payment depending on whom they were paired with for a series of games (mean payment = CHF 21.80). This study was approved by the Hautes Etudes Commerciales (HEC) Ethics Committee of the University of Lausanne.

**Table 1:** Baseline parameters and personality characteristics of all participants. Baseline cortisol: sample taken 20 min before stressor. Cognitive test: 10 min Bochumer Matrizen‐test. Personality measurements obtained with HEXACO PI‐R, except for trait anxiety measured with the STAI‐T. Differences were tested with anova. Data are presented as mean and standard deviations (SD) for each group

Observations	Control		Stress		*F*‐value	*P*‐value
	179		173			
	Mean	SD	Mean	SD		
Age	20.82	2.40	21.41	2.52	4.49	0.03
Baseline Cortisol	6.24	5.19	5.82	4.77	0.57	0.45
Cognitive test	7.63	3.09	7.57	2.61	0.05	0.82
Personality						
Honesty	33.72	7.37	32.76	7.91	1.39	0.24
Emotionality	29.88	6.82	29.20	6.56	0.90	0.34
Extraversion	34.22	6.13	35.45	5.79	3.71	0.05
Agreeableness	31.28	6.81	30.56	5.73	1.13	0.29
Conscientiousness	35.11	6.04	35.08	5.74	0.00	0.96
Openness	34.69	6.61	35.22	6.72	0.56	0.45
Trait Anxiety	33.85	10.35	31.60	8.30	5.02	0.02

### Experimental procedures

**Figure 1:**
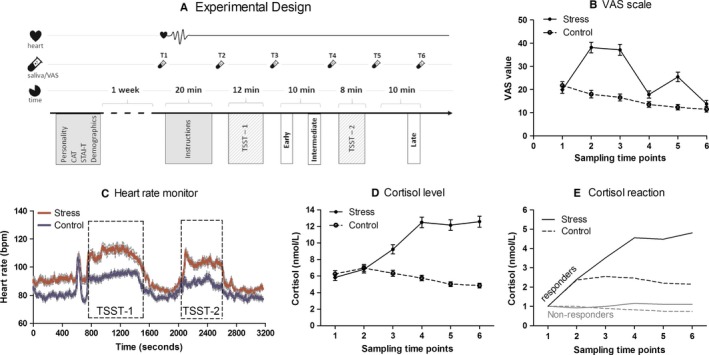
The stress induction protocol successfully induced a stress response in stress‐group subjects. Subjects performed either a TSST‐G stress procedure (TSST‐1 and TSST‐2 denote the respective interview and mathematical portions of the TSST‐G stressor) or control procedure and then performed tasks as outlined (A). Subjects in the stress group reported significantly higher levels of subjective stress during each saliva measurement (B), exhibited increased heart rates (C) and cortisol levels (D) during the experiment compared to controls. A portion of subjects exhibited a pattern of cortisol response such that they could be classified as either responders or non‐responders, with non‐responders demonstrating similar cortisol levels to control groups, and responders exhibiting significantly higher levels. Data are presented as mean and standard error, except for (E) which depicts the means. [Colour figure can be viewed at wileyonlinelibrary.com].

The procedure is outlined in Fig. 1A. One week before the experiment, participants filled out a battery of questionnaires online, including the State‐Trait Anxiety Inventory (Spielberger, 1983); and a 10‐min timed version of the Bochumer Matrizen‐test (Hossiep *et al*., 1999). Upon arrival to the laboratory, participants read and signed information and consent forms. They were then fitted with a heart rate monitor (POLAR CSX800; Polar Electro, Kempele, Finland). Saliva samples were collected using Salivette sampling devices (Sarstedt, Nümbrecht, Germany), and visual analogue scales (VAS) were given to assess subjective stress levels at different times throughout the experiment (see T1‐T6 in Fig. 1A). Economic games were explained and participants completed trial games in advance to ensure their full understanding of the tasks. Following instructions, participants were told which condition they were assigned to and were given 10 min to prepare for the interview. Participants in the stress group were exposed to the Trier Social Stress Test for Groups (von Dawans *et al*., 2011), which involves the preparation and delivery of an oral presentation simulating a job interview, as well as performing a mental arithmetic task before an unresponsive jury and video cameras. Participants in the control group were given a text to read in a low voice, followed by an easy counting task. These measures have been shown to control for different factors of the TSST‐G procedure excluding the psychosocial stress component (von Dawans *et al*., 2011). Following each of the speaking and arithmetic tasks, both groups played a series of economic games, including the standard risk and anti‐social risk games. Participants performed these two games only once. Different cohorts of control and stressed participants performed these games at different time points within the first hour after the stress induction; i.e., immediately after, and 20 and 45 min from onset. At the end of the experiment, all participants completed an attention test (Brickenkamp & Zillmer, 1998) to ensure potential differences in participants’ performance did not arise due to a lack of engagement. We verified the experiment's credibility by asking participants whether they truly believed they were matched with a live person in the anti‐social risk game, on a scale of 0 (no doubt at all) to 100 (highly doubtful) during the debriefing session. Most participants had little or no doubt (Mean = 27.05, SD = 33.24). At the end of the experiment, payoffs were calculated. Participants were paid 45 Swiss Francs (CHF 45; CHF 1 = 1.03 USD) for participation and an additional amount based on their game choices, which varied between CHF 0 and CHF 35.

### The standard risk and anti‐social risk games

**Figure 2:**
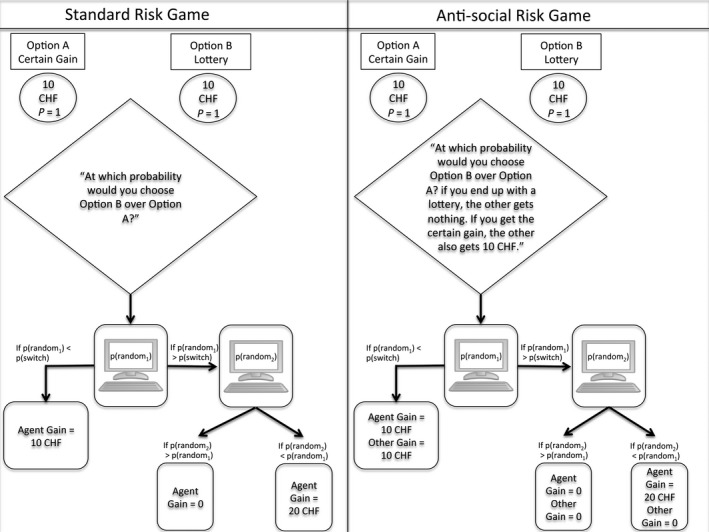
A schematic representation of the game used to assess risk preferences, under both standard risk and anti‐social conditions. Participants (denoted here as Agents) provided a switching probability ‘p(switch)’ at which they would accept a minimum probability of winning to enter a lottery for 20 CHF (Option B) over the certain gain of 10 CHF (Option A).

Participants were given two choice lottery games (see scheme in Fig. 2), one testing for individual risk (i.e., the standard risk game) and the second for other‐regarding risky behaviour (i.e., the antisocial risk game). Following the strategy method, subjects were first asked to indicate the probability *P* at which they would choose a lottery with a 20 CHF gain over a certain outcome with a 10 CHF gain (standard risk game). This measure, dubbed the switching probability threshold [p(switch)] is an indicator of risk aversion, as the higher the probability threshold, the more risk averse the subject. The game was then played a second time, whereupon a social dilemma was introduced (anti‐social risk game). While the game structure remained the same, the outcome of the decision‐making impacted a third party. When the subject obtained the certain gain, the third party also obtained 10 CHF. However, when the subject obtained the lottery, the third party obtained nothing. Thus, a pro‐social move would be to give a high p(switch), minimizing the likelihood of obtaining the lottery and its concomitant antisocial outcome. Each participant performed these two lottery games only once, corresponding with a single testing time point for each risk condition. Different cohorts of participants were tested at different time points with regards to the stress or control manipulation.

For the calculation of the payment to the participants, a computer‐generated random probability was assigned to the subject (p(random)). When p(random) was higher than p(switch), the subject obtained the payoff from a lottery with a chance of winning equal to p(random) and a gain of 20 CHF. When p(random) was smaller than p(switch), subjects were paid the 10 CHF from option A. In the anti‐social risk game, a third party was paid 10 CHF whenever the participant chose the certain gain (option A). Note that feedback regarding the results of games and payment were only given once the subjects finished all experimental procedures.

### Cortisol assessment

Saliva samples were stored at −20 °C until processed. The assay protocol was conducted as follows: samples were first centrifuged at 3000 rpm for 15 min at room temperature, then salivary cortisol concentrations were measured by enzyme immunoassay (Salimetrics, Suffolk, UK) according to manufacturer instructions. The analytical sensitivity of the cortisol assay is 0.007 μg/dL with standard curve ranging from 0.012 to 3.00 μg/dL. Coefficients of variation for low and high commercial controls were 4.75% for intra‐assay and 8.2% for inter‐assay.

### Data analysis

Data were analysed using STATA (2013, StataCorp). All simple comparisons and analyses, unless otherwise specified, were performed using between‐subjects factorial anova, and reported statistics are relative to group differences. Analyses involving covariates and interactions were performed using moderated regression with robust standard errors. Coefficients and significance levels are always reported in relevant tables for regression, and interaction terms are defined as such.

## Results

### Baseline parameters

Following recruitment, subjects were randomly distributed into control and stress groups and exposed to the experimental procedure (Fig. 1A). As shown in Table 1, although these two groups significantly differed in age and trait anxiety, mean differences between groups are very small (e.g. only 6 months difference in age), and should not represent functional differences. Otherwise, no differences in baseline cortisol, cognitive scores or psychometric variables were found between control and stressed subjects.

### Stress induction

Subjects in the stress condition gave higher subjective stress ratings on the visual analogue scale (VAS) (Fig. 1B), and showed significantly elevated cortisol (Fig. 1C) and heart rate levels (Fig. 1D) relative to participants in the control condition, indicating successful stress induction. We found no significant differences in subjective stress ratings between groups at the first time‐point, [*F*_1,226_ = 0.63, *P *=* *0.43], nor at the last [*F*_1,226_ = 1.35, *P *=* *0.25]. At all other time‐points, however, (T1–T4 in Fig. 1A), a difference in VAS ratings emerged (*F*_1,226_ = T1: 50.5, *P *<* *0.001; T2: 55.65, *P *<* *0.001; T3: 4.58, *P *=* *0.033; T4**:** 31.21, *P *<* *0.001). Similarly, salivary cortisol measures validated the stress induction procedure: no group differences were found in cortisol levels in samples taken prior to stress induction (both *F*s < 0.64, both *p*s > 0.42), however, participants in the stress condition exhibited higher levels of salivary cortisol relative to controls following stress induction (all *F*s > 19.75, all *p*s < 0.001). In addition to these analyses, participants were further split in to responder and non‐responder groups. Responders included those participants that showed a cortisol‐specific response to the stressor, defined as the ratio of cortisol at a specific time point to a baseline level. A subject was defined as a responder if the summed reactions of all time points fell at least one standard deviation above the mean reaction level of the control group. According to this criterion, 18% of controls group and 45% of participants in the stress condition qualified as responders. Figure 1E represents the average reaction for responders and non‐responders, for both control and stress groups.

Heart rate measures showed differences between control and stress groups, from the minute after the start of the measurement [*F*_1,221_ = 4.07, *P *=* *0.044] once participants received instructions for their task, further confirming that the TSST‐G procedure was effective in eliciting a physiological stress response.

### Time‐dependent effects of stress on risk aversion

**Figure 3:**
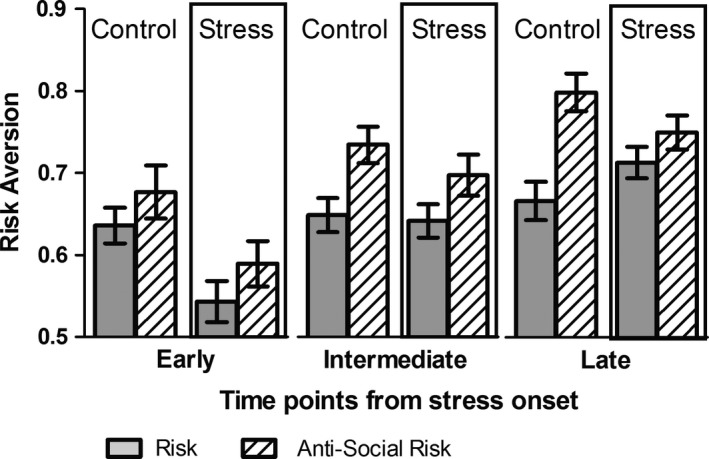
Stress caused a fluctuation of risk aversion over time that is antisocial. Switching probabilities are plotted for decisions taken at three different time points after stress induction for both stressed and non‐stressed control subjects. Higher probabilities indicate higher risk aversion. Error bars represent Standard Error of the Mean (SEM).

We then investigated whether there were time‐specific effects of stress on risk aversion. Subjects were asked to choose between a sure gain of 10 Swiss Francs (10CHF; CHF 1 = USD 1.08); or playing a lottery in which they could win 20 CHF with probability *P* or gain nothing (CHF 0) at a probability 1−*P*. Responses were collected using the strategy method: participants were asked at which probability *P* of winning 20 CHF they would choose the lottery over the sure gain. A switching probability threshold greater than 0.5 indicates risk aversion and increases in risk aversion are reflected in increases in switching probabilities. As shown in Fig. 3, there was a significant effect of stress on risk aversion. A two‐way factorial anova revealed a significant interaction between time and stress (*F*_2, 346_ = 4.92, *P *<* *0.01). Stress significantly decreased risk aversion early after stress exposure, as demonstrated by a significantly decreased switching probability in stressed subjects compared to controls (*t* = −2.90, *P *<* *0.01), but this effect of stress was absent in later decision‐making (*F*_1,226_ = 0.80, *P* = 0.35). Control subjects exhibited risk aversion that was stable over time (*F*_2,176_ = 0.86, *P *=* *0.43). A three‐way factorial anova revealed no differences between male and female participants (*F*_1,340_ = 0.75, *P *=* *0.39), and no interaction of gender with stress (*F*_2,340_ = 1.09, *P *=* *0.29) or timing (*F*_2,340_ = 0.18, *P *=* *0.83) on risk aversion.

We then asked whether cortisol responsiveness to the stress and control manipulations interacts with risk aversion. Cortisol response had a similar effect on the standard risk game, both within control and stress groups. As shown in Table 2, we performed a moderated regression to analyse the effects of time, stress and cortisol response on risk behaviour. Similar to the analysis looking at stress condition only, cortisol response (*t* = 2.09, *P *<* *0.05) and stress (*t* = 2.26, *P *<* *0.05) both interacted with time: the further away the decision point was from the stressor, the more risk‐averse the participant.

**Table 2:** The effect of time, cortisol and stress on risk aversion. Moderated regressions identified significant effects of cortisol response (Responder), stress, and interactions with time (X Timing) on risk aversion (1) and significant effects of time on anti‐social risk aversion (2). For each regression, the estimated coefficient is depicted with the standard error in parentheses below

	Model	
	Standard risk aversion	Anti‐social risk aversion
	(1)	(2)
Timing	−0.01	0.06**
Responder	−0.14*	0.08
Responder × Timing	0.06*	−0.04
Stress	−0.18*	−0.068
Stress × Timing	0.05*	0.00
Constant	0.64***	0.63***
R Squared	0.09	0.11

Significance is indicated by asterisks at the *P *< *0.05, **0.01, and ***0.001 level.

### Time‐dependent effects of stress on anti‐social risk aversion

To examine the role of stress on anti‐social risk aversion, we performed a second game in which participants were told they were matched with a randomly selected anonymous opponent also participating in the study. They were given the same choice as above; take a sure gain or play the lottery but they were also told that their decision impacted the other individual. The latter would obtain the same gain of 10 CHF should the participant choose the sure gain or get nothing should the participant opt for the lottery. They were then asked the same questions as above (i.e., at what probability *P* of winning the 20 CHF would they choose the lottery over the sure gain). In this scenario, higher switching probability thresholds indicate more other‐regarding behaviour and vice versa. A two‐way factorial anova revealed a significant negative effect of stress on anti‐social risk aversion on switching probabilities (*F*_1,346_ = 7.39, *P *<* *0.01), a general effect of time on anti‐social risk aversion (*F*_2,346_ = 18.54, *P *<* *0.001) but no difference of the effect of stress across time (*F*_2,346_ = 0.43, *P *=* *0.65). Thus, when risk encompassed an anti‐social component, stressed participants did not modify their behaviour to take the other person into consideration as much as control subjects do. Control subjects, however, significantly increased their switching probabilities over time (*F*_2,176_ = 7.17, *P *=* *0.001), suggesting a decrease in anti‐social risk behaviour in the later time points. As with the previous game, a three‐way factorial anova revealed no difference between male and female participants (*F*_1,340_ = 0.04, *P *=* *0.84), and no interaction with time (*F*_2,340_  = 0.84, *P *=* *0.43) or stress (*F*_1,340_ = 0.25, *P *=* *0.61).

### Effects of stress on cortisol responders

In the standard individual lottery game, there is an influence of both the experimental manipulation and the cortisol response on risk aversion. The stressor and the cortisol have a similar effect on behaviour. Thus, those subjects who showed a salivary cortisol response exhibited a stronger behavioural reaction than subjects who were not responsive. The smaller fraction of controls that exhibited a cortisol response also displayed the same pattern of altered behaviour than those in the treatment condition (*t* = −2.00, *P* < 0.05, Table 2, model 1). Non‐responders in the stress condition exhibited the same reaction as responders in the control condition (Wald test, *F*_1, 330_ = 0.00, *P* = 0.96).

Responders and stressed participants had lower switching probability thresholds, thus indicating lower risk aversion, but this effect dwindled with time. In the case of the anti‐social lottery, multiple regression only shows an effect of time on anti‐social behaviour, but no further effect of cortisol response or stress on behaviour (Table 2, model 2).

### Individual differences in risk aversion and their interaction with stress

A moderated regression was performed to analyze the effect of both cognitive ability test (CAT) and anxiety on both standard risk‐aversion and anti‐social risk aversion. Table S1 shows the result of the four regression models. Models 1 and 2 study the effect of the Cognitive Test on standard risk aversion and anti‐social risk aversion, respectively, and models 3 and 4 address the effect of anxiety. CAT results significantly predicted risk aversion (*t* = 2.96, *P *<* *0.01), although this effect dwindled with time (interaction of time and CAT: *t* = −2.46, *P *<* *0.05). There were no effects or interactions of anxiety on standard risk (*t* = 0.93, *P* = 0.35), interaction of stress and anxiety: *t* = −0.37, *P* = 0.71), or anti‐social risk behaviours (*t* = −0.41, *P* = 0.68, interaction of stress and anxiety: *t* = −0.08, *P* = 0.93). Table S2 shows that the main results discussed are robust when the regressions are run using a large series of covariates, including age, gender and personality.

## Discussion

Stress is a complex phenomenon involving multiple physiological, behavioural and cognitive adaptations that follow dynamic time‐dependent patterns. Although the neurobehavioural sciences tend to consider the aftermath of acute stress exposure as a homogeneous period in terms of its modulatory influences, in fact, the first post‐stress hour is rather multidimensional. This is clearly illustrated by the changing pattern of activation typically exhibited by the SNS and HPA axis during the first hour following stress exposure. Specifically, at the peripheral level, a prototypic stress response consists of an initial transient predominance of SNS activation followed by a gradual increase in bloodstream cortisol levels that peak around 15–30 min post‐stress, and then steadily decline (van den Bos *et al*., 2013a). In the brain, different acute stress‐induced waves of neurochemical changes (Joels & Baram, 2009) are believed to correspond to differential regulation of multiple functional networks (Hermans *et al*., 2014). This dynamic picture of different neurophysiological states could engender different cognitive dispositions. Our study supports this view by showing that decision‐making processes are differentially affected at three different time points within the first hour following exposure to psychosocial stress (immediately after, 20, and 45 min from stress onset). Importantly, risk preferences under stress are selfish but follow a steady slope from reduced to enhanced risk aversion in the course of half‐an‐hour.

Therefore, a major finding in our study is that risk aversion, as evaluated in the gain domain, shows a time‐dependent change across the first post‐stress hour: shortly after stress, individuals are less risk averse for gains, an effect that progressively vanishes with time, switching towards more risk averse behaviour at the 45 min post‐stress time point. Thus, risky decisions following stress exposure show a positive slope in which behaviour turns in opposite directions; i.e., from a first reduction to a subsequent increase in risk‐averse choices. In the immediate aftermath of stress, individuals are more risk taking (i.e., less risk averse) despite the advantage provided by a risk avoidance strategy, which is in line with the proposed evolutionary value of the ‘flight or fight’ response (Starcke & Brand, 2012). At this early time point, there is a prevalence of SNS activation. Later in time, as cortisol responses increase, risk aversion is established. Importantly, cortisol responding showed the same effects as stress in risk taking, with high‐responder subjects displaying the described fluctuating changes in risk aversion with time.

Our findings hold important implications for understanding discrepancies in the literature surrounding the effects of acute stress on risk and antisocial behaviour. For example, at first glance, our results seem to be at odds with those of von Dawans *et al*., who did not find evidence of changes in nonsocial risk taking after exposure to the same stressor as in our study (von Dawans *et al*., 2012). However, in their study, different variants of the game were played several times within 15–30 min from stress onset and results averaged across different time points, which does not evaluate potential time‐dependent differences. In studies that measured behaviour shortly following stress (Starcke *et al*., 2008; Lighthall *et al*., 2009; Porcelli & Delgado, 2009; Buckert *et al*., 2014), risk aversion was decreased in stressed subjects, thus supporting our findings and underscoring the importance of factoring time into behavioural measurements. Moreover, games in the von Dawans *et al*. (2012) study did not include the possibility to be antisocial. Thus, our studies measure different types of preference. However, in the only study that, to our knowledge, has assessed decision‐making at several time points following exposure to stress (TSST; note that this was performed in isolation, not in groups as in our study), more risk aversion was observed in the immediate aftermath of stress that became riskier when subjects were tested 28 min from stress onset (Pabst *et al*., 2013b). The discrepancy with our results is probably due to the different nature of the games [the Game of Dice (Starcke *et al*., 2008)] used in the respective studies. Particularly, our subjects were only presented with choices for gains, while in the Pabst *et al*. study each choice engendered gains and losses (Pabst *et al*., 2013b). This is a key difference, as the domain/s engaged in economic choices (i.e., gain, loss, or both) are known to affect decision‐making (Kahneman & Tversky, 1979). In fact, higher risk aversion was also reported by another study that tested participants for choices related to gains and losses following exposure to a brief stressor (Porcelli & Delgado, 2009; cold pressor combined with memory task). In support of our interpretation, no effect of psychosocial stress in the Game of Dice was found in the gain domain when participants were only given choices for gains and tested 10 min post‐stress (Pabst *et al*., 2013b), a finding that fits with the lack of effects observed in our study at that time point. Moreover, some of the studies reporting results discrepant to ours (Pabst *et al*., 2013a) included several trials through which participants received feedback about gains and losses, a learning component that is absent in our experiment. Importantly, when feedback was not provided and individuals were tested immediately (0–15 min) after stress exposure, other studies found more risk taking for gains using financially incentivized lotteries similar to ours; however, the effects were sometimes only apparent for subjects that showed a robust cortisol response (Buckert *et al*., 2014). Our findings identifying high cortisol responder subjects as particularly affected in their risk taking behaviour with time are in agreement with these and other studies that also found riskier (van den Bos *et al*., 2009) and less strategic (Leder *et al*., 2013) behaviours in subjects showing high cortisol responses to psychosocial stress.

Our second main finding is that stress led to selfish decisions: stressed individuals focused on their own choices and neglected the negative consequences to other social agents. This effect was globally present across all testing times, as stressed subjects were significantly more likely to make risky decisions when the outcome was antisocial. Across time, choices from control subjects progressively took into account the anti‐social consequence of choosing to play the lottery when a second subject was involved (remember that the second player would only receive earnings if the participant chose the sure gain, and nothing when choosing the lottery), correcting their decisions towards a higher preference for the certain gain, rather than the lottery. However, stressed subjects appeared locked in their own decisions, as they did not modify their choices to account for the negative consequences to the other player. These observations are in line with emerging evidence indicating that stress can have deep effects on social behaviours (Sandi & Haller, 2015). However, in this case, cortisol did not explain stress effects in the anti‐social choice game which conflicts with studies in animals implicating glucocorticoids in stress‐induced aggressive behaviour (Haller, 2014). It is important to note that the degree of stress experimentally induced in the animal literature is typically well beyond the one recreated in human experimental settings. Accordingly, we cannot exclude the anti‐social influence of glucocorticoids in humans under circumstances involving higher cortisol levels or actual social confrontations.

Our behavioural results in the anti‐social game contrast with previous studies in which stressed male participants showed increased pro‐social behaviours, including elevated levels of generosity when making decisions (von Dawans *et al*., 2012; Margittai *et al*., 2015). It is important to note that in those studies participants were directly and explicitly asked to decide whether to give money to another subject. On the contrary, in our study, the decision regarding the other subject is implicit. Specifically, participants were requested to make a choice regarding their preference on a risk game with consequences for themselves and told that their choice would have monetary consequences on another subject. Moreover, whereas our study allowed participants to ponder their preferences for risk across a full range of risk taking options, games in other studies (von Dawans *et al*., 2012) included binary choices (e.g., trustworthiness or no trustworthiness, sharing or no sharing). Furthermore, detailed analyses of the reported stress‐induced generosity indicated that it emerges only when *socially close individuals* are affected in a modified version of the Dictator game shortly after stress exposure (Margittai *et al*., 2015; TSST‐G). In our study, the ‘other’ was anonymous and unfamiliar, and hence fits with lower generosity reported in stressed subjects when a donation was given to a charitable organization (Vinkers *et al*., 2013). Interestingly, this effect in trust behaviour (measured with the Ultimatum game) reported by Vinkers *et al*. was time‐dependent as it was observed immediately after stress exposure, but not 75 min later (Vinkers *et al*., 2013). Additionally, in line with our findings, Starcke *et al*. also found more egoistic decision‐making when confronted with social dilemmas in participants that were tested in the immediate aftermath of social stress (TSST; Starcke *et al*., 2011).

Control subjects in our study showed persistent signs of risk aversion (scores around 0.65), which is in agreement with behaviour observed in humans and many other species^,^ (Caraco *et al*., 1980; Barkan, 1990). However, and surprisingly, their other‐regarding decision‐making showed a progressive change across the three testing times. Although by the second and particularly the third time points, control subjects were willing to forego lotteries with higher winning probabilities to take into account the anti‐social consequences of playing the lottery, this behaviour was not observed when they played the game shortly after the ‘control’ manipulation. Although this effect was somewhat unexpected, there are possible explanations that could account for this differential behaviour when behaviour is measured at different time points following the ‘control’ manipulation. In the control manipulation, subjects had to read a text followed by an easy counting task, all in a low voice and, as opposed to the stress manipulation in which subjects performed out loud and each participant spoke one at a time, subjects in the control group did their reading and counting simultaneously. However, the fact that subjects in the control group had to perform these tasks in close proximity to the other participants (note that they were tested in groups of six) and in front of a jury, even if ‘friendly’, is likely to induce mild arousal. An indication for this interpretation seems to be the mild increase in heart rate observed in Fig. 1C from baseline to the control manipulation, which might not only reflect changes elicited by changing position from sitting to standing but also a certain arousal. In fact, there is evidence that controls subjected to the same experimental procedures display increased markers of SNS (e.g., increased heart rate, increased salivary alpha‐amylase, which is under adrenergic control and therefore an indirect marker of SNS activity) activation shortly after ‘control’ manipulations in the TSST, but not at later time points (Pabst *et al*., 2013a; Vinkers *et al*., 2013). This suggests that, during the early testing time point, control subjects responded under increased arousal and sympathetic activation – supposedly paralleled by increased brain noradrenergic activation – and might explain why at this, but not later time points (note that the control group do not mount a cortisol response as observed in the stress group), they did not ‘correct’ their decisions to take into account the consequences to the other subject, resembling the pattern observed in stressed subjects. In addition, it is worth noting that control subjects in our study had higher trait anxiety levels than their experimental counterparts. Although the difference was rather small, we cannot discard its potential influence on subjects’ reactivity immediately after exposure to the ‘control’ manipulation. However, note that, overall, the reported results in the two games regarding the effects of stress did not differ when anxiety was treated as a covariate.

Regarding individual differences, we found no effects of gender or anxiety on self or anti‐social risk‐taking. The lack of gender effects in risk‐taking are surprising, as former studies found that, as opposed to males, females become more risk‐averse following stress exposure (Preston *et al*., 2007; van den Bos *et al*., 2009; Lighthall *et al*., 2009; Mather & Lighthall, 2012). Similarly, gender has been proposed to be an important modulatory factor in the social impact of stress, with females’ responses following a pattern of “tend‐and‐befriend”, whereas a “fight‐or‐flight” pattern is pursued in both males and females (Taylor *et al*., 2000), although the recent literature has not always validated this distinction (von Dawans *et al*., 2011). Our data does not confirm a gender distinction for risk and anti‐social risk responding under stress; however, a lack of statistical power might be responsible for this absence of gender effects. In addition, our study found that high cognitive scores as assessed by the CAT test predicted performance in the games (higher scores corresponded to higher risk aversion) but did not interact with stress effects on either game. These findings are somehow at odds with emerging evidence indicating that individuals that score higher in tests for executive function are less vulnerable to performance deficits in cognitive tasks resulting from stress or anxiety (Johnson & Gronlund, 2009; Owens *et al*., 2014; Edwards *et al*., 2015; Thoresen *et al*., 2016).

Time‐dependent effects in behaviour and cognition occurring from the minutes to hours following exposure to stress have been previously highlighted, with the temporal distinction mainly placed between two time points, one occurring within the first post‐stress hour with an engagement of brain noradrenergic mechanisms and non‐genomic corticosteroid effects and the second one from about 1–4 h post‐stress and corresponding to genomic corticosteroid effects (Hermans *et al*., 2014). Two receptors are involved in corticosteroid actions: the mineralocorticoid (MR) and the glucocorticoid (GR) receptors. Recent integrative models of stress actions on cognition (de Kloet *et al*., 2016; Vogel *et al*., 2016) propose a key role for the MR in mediating the rapid behavioural, cognitive, and neural adaptations that follow exposure to acute stress, supposedly in close interaction with the known rapid activation of catecholamines (Arnsten, 2009), and a subsequent engagement of the widely distributed lower affinity glucocorticoid receptor (GR) involved in subsequent management of stress adaptation (de Kloet *et al*., 2009). Thus, within the post‐stress time window considered in our study, the predominant rapid brain mechanisms are supposed to engage noradrenergic‐ and MR‐mediated mechanisms, engaging a salience network (Hermans *et al*., 2014) and a shift towards cognitively less‐demanding processing and allowing a quick response to a situation (Vogel *et al*., 2016). These processes have been proposed to occur at the cost of an executive control network, which will be activated in a second temporal window normalizing emotional reactivity and enhancing higher‐order cognitive processes (Hermans *et al*., 2014). Thus, rapid actions taking place immediately after stress exposure have been shown to engage striatal pathways (Schwabe & Wolf, 2013; Vogel *et al*., 2016), which might correspond with the increased interest for the incentivized lottery observed in our study in the immediate aftermath of stress. In addition, the noradrenergic activation taking place at this early time point may also play a role in modulating more selfish behaviour. Indeed, noradrenergic blockade has been shown to decrease utilitarian judgment (Terbeck *et al*., 2013). However, a limitation of our study is the fact that our experimental design included a potentially confounding factor regarding stress timing. We followed previously published TSST‐G procedures involving two stress induction blocks (see Fig. 1A; von Dawans *et al*., 2011, 2012; Goette *et al.,* 2015) which implies that participants tested in the late testing time in our study were not only tested late from stress onset but also following an additional and recent stress induction procedure. Although these observations imply that we should consider our late time‐dependent effects of stress with caution, the linear pattern observed for the responses to the standard risk game with time and the sustained effect of stress in the anti‐social risk game across the different testing times supports the validity of our conclusions.

Importantly, our data reporting time‐dependent effects occurring at three different time periods within the first post‐stress hour argues for the need to redefine dynamic mechanisms occurring within this period. Therefore, our findings argue for the need to investigate the dynamic pattern of neural dynamics at different time points within the first post‐stress hour in order to better understand the correspondence between the progressing pattern of neurobiological processes triggered by stress and the flexibly allocated behavioural and cognitive adaptations.

## Conflict of interests

The authors declare no competing financial interests with respect to authorship or the publication of this article.

## Author contributions

S.B., L.G. and C.S. conceived the study and designed the methodology. J.T. and L.L.‐K. performed the experiments. S.B., L.G., F.H. C.S. performed the analyses of the experimental data. C.S. wrote the article with contributions and input from S.B., L.G., L.L.‐K. and F.H.

**Abbreviations**
CAT: cognitive ability test
CHF: Swiss Francs
HPA: hypothalamic‐pituitary‐adrenal
Min: minute
SNS: sympathetic nervous system
STAI: state trait anxiety inventory
TSST‐G: Trier Social Stress Test for Groups
TSST: Trier Social Stress Test

## Supporting information

Table S1 Individual differences in cognitive ability and trait anxiety on risk taking.
Table S2 Test of robustness of main results when including a various set of covariates.
