# Supplementary material for: Acute stress alters individual risk taking in a time‐dependent manner and leads to anti‐social risk
Source: Eur J Neurosci. 2016 Sep 23;45(7):877–85. doi: 10.1111/ejn.13395 (PMC5396300; doi:10.1111/ejn.13395)
Supplement: Supplementary file 1 — Table S1 Individual differences in cognitive ability and trait anxiety on risk taking. Table S2 Test of robustness of main results when including a various set of covariates. [file EJN-45-877-s001.pdf]

Supplementary Material

Acute stress alters individual risk taking in a time-dependent manner and leads to anti-social risk

Bendahan S.[1,2]*, Goette L.[2*#], Thoresen J.[1], Loued-Khenissi L.[1], Hollis F.[1], Sandi C.[1]*

[1]Laboratory of Behavioral Genetics, Brain Mind Institute, Ecole Polytechnique Fédérale de Lausanne (EPFL), CH-1015 Lausanne Switzerland

[1]Department of Economics, Faculty of Business and Economics, University of Lausanne (UNIL), CH-1015 Lausanne, Switzerland

We investigated the role executive function – as assessed by a cognitive test (CAT) – may play in modulating the effects of stress on risk behavior. We found that CAT scores correlate with increased risk aversion both in standard risk and anti-social risk conditions (Table S1). Additionally, as we had a significant difference in trait anxiety between our control and stress groups, we examined whether trait anxiety modulated the effects of stress on risk behavior. As shown in models 3 and 4 in Table S1, we found no significant effects of anxiety or interactions on risk behavior.

Estimated coefficient	CAT analysis		Anxiety analysis	
	Standard risk aversion	Anti-social risk aversi	Standard risk aversior	Anti-social risk aversion
	(1)	(2)	(3)	(4)
Stress	-.21*	-0.11	-.553*	-0.3
Timing	0.088*	0.158***	-0.084	-0.03
Cat	0.029**	0.034**		
Anxiety			-0.005	-0.07
Interactions				
Stress X Timing	0.07**	0.018	.189*	0.08
Stress X Cat	-0.001	0.000		
Timing X Cat	-0.009*	-0.012*		
Stress X Anxiety			0.006	0.003
Timing X Anxiety			.003	0.003
Constant	0.39***	0.35***	.796***	0.82***
R Squared	0.13	0.15	0.1	0.13

Table S1. Individual differences in cognitive ability and trait anxiety on risk taking. Regression models examine the effects of cognitive ability test performance (CAT) and trait anxiety on risk and anti-social risk aversion. Significance is indicated by asterisks at the p< *.05, **.01, and ***.001 level.

We also investigated whether our main results still hold when including age, sex, cat and personality as covariates.

Estimated coefficients	Model			
	Standard Risk	Standard Risk	Anti-social Risk	Anti-social Risk
	(1)	(2)	(3)	(4)
Stress	-0.23***	-0.241***	-0.11	-0.149
	(0.072)	(0.073)	(0.08)	(0.088)
Timing	0.02	0.0173	0.07***	0.066
	(0.016)	(0.016)	(0.02)	(0.02)
Stress X Timing	0.07**	0.074**	0.018	0.03
	(0.023)	(0.023)	(0.03)	(0.028)
Covariates				
Age		0.0014		0.0047
		(0.004)		(0.005)
CAT		0.012***		0.013**
		(0.004)		(0.004)
Anxiety		0.0003		-0.0014
		(0.001)		(0.001)
Male		-0.011		0.016
		(0.022)		(0.026)
Honesty		-0.0019		0.000
		(0.001)		(0.002)
Emotionality		0.0028		0.004*
		(0.002)		(0.002)
Extraversion		0.0024		0.003
		(0.002)		(0.002)
Agreeableness		0.001		0.0014
		(0.002)		(0.002)
Conscientiousness		0.003		0.0018
		(0.002)		(0.002)
Openness		-0.002		-0.0027
		(0.002)		(0.002)
Constant	0.615***	0.34*	0.605***	0.188
	(0.034)	(0.171)	(0.040)	(0.206)
R Squared	0.09	0.177	0.12	0.184

Table S2: test of robustness of main results when including a various set of covariates. Regression models examine the effects of stress and timing on standard risk and anti-social risk game. Significance is indicated by asterisks at the p< *.05, **.01, and ***.001 level.
